# Monthly Summary

**Published:** 1890-01

**Authors:** 


### Monthly Summary.

CONGENITAL SYPHILIS IN INFANTS.—The next case shown was of especial intent on account of its serious nature, comparative frequency, and the importance of making a correct diagnosis. The infant was healthy at birth, but in a week it had the snuffles. Soon afterwards it exhibited on head, trunk, and extremities copper colored spots, especially about the face and genitalia. It was remarkable that there are three grades or degrees of congenital syphilis. In the first the child is born dead. In the second class it is born in a miserable condition of deformity and disease, and is soon carried off by fibroid pneumonia lack of vitality, or some disease of an important viscus. In the third class the child is born apparently healthy, and the disease first appears some days or weeks after birth, as in this case, That it is often well in such cases not to question either parent too closely, but to make the diagnosis by the child's appearance, and to proceed at once with the following line of treatment.

After carefully seeing to its hygienic management, the infant is to have twice a day one-sixteenth to one-twelfth of a grain of calomel in a little soda; or, if the child cannot from any reason take calomel internally, mercurial ointment is to be rubbed on the child's binder, which is then applied. In a few weeks the spots will disappear and the child seem well for a while. In order to completely eradicate the disease from the system, mercury and the iodide of potassium are to be skillfully administered with occasional breaks in the treatment for two or three years. The liver is subject to congestion in these cases, and such an infant should never be nursed by any woman but its own mother, lest the disease be communicated to her.—*Phila. Hospital Reports in Medical and Surgical Reporter.*

---

SOURCE OF COLORS.—The cochineal insects furnish a great many colors. Among them are carmine, crimson, scarlet carmine, and purple lakes. A sea shell belonging to the purpura, and found in Japanese waters, gives a rich violet dye. The cuttlefish gives the sepia. It is the inky fluid which the fish discharges in order to render the water opaque when attacked. Indian yellow comes from the feces of the camel. Ivory chips produce ivory black and bone black. Prussian blue is made by fusing horses' hoofs and other refuse animal matter with impure potassium carbonate. Various lakes are derived from roots, barks, and gums. Lamp black is soot from certain resinous substances. Turkey red is made from the madder plant, which grows in Hindostan. The yellow sap of a tree of Siam produces gamboge; the natives catch the sap in cocoanut shells. Raw sienna is the natural earth from the neighborhood of Sienna, Italy. Raw umber is also an earth found near Umbria and burnt. India ink is made from burnt camphor and gum. The Chinese and Japanese are the only manufacturers of this ink. The process is a tedious one and requires great skill. The finer grades of India ink are delicately scented with attar of roses, and one stick about three inches long may cost four or five dollars. Age improves the ink. Mastic is made from the gum of the mastic tree, which grows in the Grecian Archipelago. Bistre is the soot of wood ashes. Very little real ultramarine is found in the market. It is obtained from the precious lapis-lazuli, and commands a fabulous price. Chinese white is zinc, scarlet is iodide of mercury, and native vermilion is from the quicksilver ore called cinnabar.—*Medical and Surgical Reporter.*

